# COVID-19 in Patients with Diabetes: Clinical Course, Metabolic Status, Inflammation, and Coagulation Disorder

**DOI:** 10.17691/stm2020.12.5.01

**Published:** 2020-10-28

**Authors:** D.V. Belikina, E.S. Malysheva, A.V. Petrov, T.A. Nekrasova, E.S. Nekaeva, A.E. Lavrova, D.G. Zarubina, K.A. Atduev, D.M. Magomedova, L.G. Strongin

**Affiliations:** PhD Student, Department of Endocrinology and Internal Medicine; Privolzhsky Research Medical University, 10/1 Minin and Pozharsky Square, Nizhny Novgorod, 603005, Russia;; Assistant, Department of Endocrinology and Internal Medicine; Privolzhsky Research Medical University, 10/1 Minin and Pozharsky Square, Nizhny Novgorod, 603005, Russia;; Associate Professor, Department of Endocrinology and Internal Medicine; Privolzhsky Research Medical University, 10/1 Minin and Pozharsky Square, Nizhny Novgorod, 603005, Russia;; Professor, Department of Endocrinology and Internal Medicine; Privolzhsky Research Medical University, 10/1 Minin and Pozharsky Square, Nizhny Novgorod, 603005, Russia;; Head of the Emergency Department; Infection Hospital for Patients with Novel Coronavirus Infection (COVID-19) at the Institute of Pediatrics of the University Hospital, Privolzhsky Research Medical University, 22, Bldg 1, Semashko St., Nizhny Novgorod, 603950, Russia; Head of the Department of Pediatrics; Infection Hospital for Patients with Novel Coronavirus Infection (COVID-19) at the Institute of Pediatrics of the University Hospital, Privolzhsky Research Medical University, 22, Bldg 1, Semashko St., Nizhny Novgorod, 603950, Russia; Student, Medical Faculty;Privolzhsky Research Medical University, 10/1 Minin and Pozharsky Square, Nizhny Novgorod, 603005, Russia;; Student, Medical Faculty;Privolzhsky Research Medical University, 10/1 Minin and Pozharsky Square, Nizhny Novgorod, 603005, Russia;; Student, Medical Faculty;Privolzhsky Research Medical University, 10/1 Minin and Pozharsky Square, Nizhny Novgorod, 603005, Russia;; Professor, Head of the Department of Endocrinology and Internal Medicine Privolzhsky Research Medical University, 10/1 Minin and Pozharsky Square, Nizhny Novgorod, 603005, Russia;

**Keywords:** COVID-19, SARS-CoV-2, diabetes mellitus, glycemia, hypercoagulation, systemic inflammation

## Abstract

**The aim of the investigation** was to study the clinical course of COVID-19 in the presence of diabetes mellitus (DM) and elucidate possible mechanisms of their mutual aggravation.

**Materials and Methods.:**

The study included 64 patients with COVID-19; of them, 32 were with DM (main group) and 32 were DM-free (control group). The groups were formed according to the “case–control” principle. During hospitalization, the dynamics of clinical, glycemic, and coagulation parameters, markers of systemic inflammation, as well as kidney and liver functions were monitored and compared.

**Results.:**

Among patients with DM, the course of viral pneumonia was more severe, as evidenced by a 2.2-fold higher number of people with extensive (>50%) lung damage (p=0.05), an increased risk of death according to the CURB-65 algorithm (1.3-fold, p=0.043), and a longer duration of insufficient blood oxygen saturation (p=0.0004). With the combination of COVID-19 and DM, hyperglycemia is persistent, without pronounced variability (MAGE — 1.5±0.6 mmol/L), the levels of C-reactive protein (p=0.028), creatinine (p=0.035), and fibrinogen (p=0.013) are higher, manifestations of hypercoagulability persist longer, including slower normalization of antithrombin III (p=0.012), fibrinogen (p=0.037), and D-dimer (p=0.035).

**Conclusion.:**

The course of COVID-19 in patients with DM is associated with a high severity and extension of pneumonia, persistent decrease in oxygen supply, high hyperglycemia, accelerated renal dysfunction, systemic inflammation, and hypercoagulability.

## Introduction

Epidemiological studies carried out during the current COVID-19 pandemic demonstrate a powerful negative effect of comorbid pathology on the severity and outcomes of SARS-CoV-2 viral infection [1–6].

Cardiovascular diseases, arterial hypertension (AH), and diabetes mellitus (DM) are most common conditions found among patients diagnosed with COVID-19. According to the experience of pandemic-affected countries and communities, these comorbidities are associated with the maximal number of complications in COVID-19 infected patients [2–6].

Thus, according to the observations of Chinese scientists, most deaths occurred among patients with comorbid pathology, including AH (53.8%), DM (42.3%), heart disease (19.2%), and strokes (15.4%) [5]. In Italy, the most severely ill patients requiring treatment in the intensive care unit often had AH (49%), other cardiovascular diseases (21%), DM (17%) [7]. Prevalence of DM among the deceased people infected with SARS-CoV-2 was 35.5% [8]. In the USA, among patients with COVID-19, DM was detected in 10.9%, and among those in need of treatment in the ICU — in 32% of cases [9].

According to a recent meta-analysis [10], when COVID-19 is combined with DM, the risk of composite adverse outcome increases (relative risk, RR — 2.38 [1.88; 3.03]; p<0.001), including mortality (RR — 2.12 [1.44; 3.11]; p<0.001), severe course of COVID-19 (RR — 2.45 [1.79; 3.35]; p<0.001), acute respiratory distress syndrome (RR — 4.64 [1.86; 11.58]; p=0.001), and disease progression (RR — 3.31 [1.08; 10.14]; p=0.04) [10]. Obesity, which often occurs in DM, also contributes to the worsening prognosis [11].

These results suggest a significant role of DM in the development of severe clinical forms and mortality in COVID-19. Since DM is often associated with other risk factors like hypertension and other cardiovascular diseases, obesity and old age, such patients require special approaches to their prognosis and treatment strategy.

For the successful treatment of patients with COVID-19 combined with DM, it is important to know the mechanisms that mediate the aggravated course of the combined disease. It is also important to identify predictors of adverse outcomes in patients with a combination of COVID-19 and DM in order to timely choose the optimal management tactics for such patients.

There are different views in the literature regarding the mechanisms underlying the severity of SARS-CoV-2 infection complicated by DM. A significant role can be played by inflammatory changes and immunity disorders characteristic of DM, including suppression of neutrophil chemotaxis and T lymphocyte-mediated immune response, impaired cytokine production, and decreased elimination of pathogens [12–16], including SARS-CoV-2 [17]. The DM-associated obesity adds to systemic inflammation in two ways. Firstly, an excess of adipose tissue contributes to inflammation by increasing the production of pro-inflammatory cytokines, adipokines, and chemokines. Secondly, obesity is associated with a deficiency of vitamin D — an inhibitor of inflammatory processes. Both mechanisms can increase the severity of COVID-19 [18].

Another potential point of intersection between DM and COVID-19 is the expression of angiotensin-converting enzyme 2 (ACE2). This enzyme serves as a functional component of the renin-angiotensin-aldosterone system (RAAS): while ACE converts angiotensin I into angiotensin II, ACE2 converts angiotensin II into angiotensin 1–7. In this process, the vasoconstricting and pro-inflammatory effects of angiotensin II are balanced by the vasodilating and anti-inflammatory properties of angiotensin 1–7 [19, 20]. The role of ACE2 in COVID-19 is twofold: on the one hand, it is the site of binding of SARS-CoV-2 to the cell, and on the other hand, a low expression of ACE2 aggravates the lung damage caused by the infection [20]. Specific mechanisms of aggravated RAAS disorders in the COVID-19/DM combination may be associated with: 1) a decrease in the ratio of ACE2/ ACE in the lungs at late stages of DM [21], which under COVID-19 does not rule out a unidirectional negative effect on the balance of angiotensins; 2) the influence of ACE2 expression in pancreatic β-cells on their function, which suggests the possibility of hyperglycemia in COVID-19 [22, 23]; 3) an impaired endothelial function caused by a direct contact between the virus and ACE2 on the surface of endothelial cells [24]; 4) frequent cases of using RAAS inhibitors to treat hypertension in patients with DM [25, 26].

Liver dysfunction [27, 28], coagulation disorder [29], and endothelial dysfunction [24] in patients with diabetes are considered as major aggravating factors in COVID-19.

More data on the interaction between COVID-19 and DM are needed to predict complications and initiate timely and adequate pathogenetic therapy in this large cohort of patients. To that end, an in-depth study of the course of COVID-19 in patients with DM including the local specifics and the common patterns of the disease is vital.

Considering the above, we **aimed** to study the clinical course of COVID-19 in the presence of DM and elucidate possible mechanisms of their mutual aggravation.

## Materials and Methods

This open comparative study was conducted in 64 patients with COVID-19 hospitalized at the University Hospital of the Privolzhsky Research Medical University from May 19 to June 21, 2020; of those patients, 32 people were diagnosed with DM (main group). The study was conducted in accordance with the Declaration of Helsinki (2013) and approved by the Ethics Committee of the Privolzhsky Research Medical University. Informed consent was obtained from each patient. The first stage of the study was cross-sectional, when the actual clinical characteristics of patients with and without DM were compared at the time of admission. The second stage was prospective in nature and included dynamic assessments of clinical indicators and outcomes in the compared groups. The occurrence of the composite endpoint (ICU treatment and/or death) was considered an unfavorable outcome.

The main criteria for inclusion in the study for all patients were: 1) a positive laboratory test for SARS-CoV-2 (by swab from the nasopharynx and oropharynx); 2) CT signs of viral pneumonia.

Criteria for inclusion in the main group were: 1) type 2 DM diagnosed by medical history, glycemic profile and glycated hemoglobin (HbA1c) exceeding the normal values in most patients; or 2) DM newly diagnosed from a characteristic glycemic profile and an increase in HbA1c at the time of hospitalization.

The control group (COVID-19 without DM) was created according to the “case–control” principle: after the inclusion of a patient with DM in the main group, the control was added with a hospitalized patient without DM of the same sex and age group.

As a result, the control and main groups were comparable in gender (10 males — 31.2%), age (56.1±13.8 and 60.4±12.0 years), and body mass index (31.8±5.5 and 33.7±6.9, respectively; p>0.05 for all indicators).

Among concomitant diseases, AH was most often detected, and its prevalence was significantly lower in the control compared with the main group — 10 (31.2%) and 20 (62.5%) patients, respectively, p=0.012. In addition, there was a trend towards a higher incidence of CHD among patients with DM — 8 (25.0%) vs 3 (9.4%), p=0.09. The groups did not differ in the occurrence rates of other comorbidities, including chronic liver diseases — 4 (12.5%) and 3 (9.4%), lung diseases — 2 (6.2%) and 1 (3.1%), and kidney diseases — 1 (3.1%) and 2 (6.2%); p>0.05 for all indicators.

In the main group, there were 19 patients (59.4%) with newly diagnosed DM and 13 patients (40.6%) with previously diagnosed DM2. In patients with a history of DM, the duration of diabetes was 3.7±5.9 years; in most cases, a diagnosis of diabetic polyneuropathy was made before this hospitalization (12 out of 13 patients). In the subgroup of patients with newly diagnosed DM, there were no individuals with manifestations of microangiopathies. Glycated hemoglobin in patients of the main group averaged at 8.3±1.6% (9.1±1.9% in patients with previously diagnosed and 7.9±1.3% in patients with newly diagnosed DM; p=0.062).

Glucose-lowering therapy was prescribed in accordance with the recommendations of the National Medical Research Center of Endocrinology of the Ministry of Health of Russia [30]; 14 people (48.8%) received insulin; of the oral medications, sulfonamides were used most often (11 patients, 34.4%). Treatment for SARS-CoV-2 infection is detailed in Results and Discussion.

The severity of pneumonia and the likelihood of death were evaluated using a modified CURB-65 scale, the nature and volume (percent) of lung tissue damage — by CT, blood oxygen saturation (SpO_2_) — by pulse oximetry. Hematological and biochemical indices, C-reactive protein (CRP) were measured using standard techniques. Aspartate aminotransferase (AST) and alanine aminotransferase (ALT) were assayed according to Reitman–Frankel, hemostasis parameters (D-dimer, INR, antithrombin III (AT III) activity, prothrombin time (PTT), activated partial thromboplastin time (APTT)) — by coagulometry using an ACL Elite Pro analyzer (Instrumentation Laboratory, USA).

The glycemic profile in patients with diabetes was studied by repeated measurements of glucose levels using a desktop analyzer (9 measurements per day) and the levels of glycated hemoglobin HbA1c — using a NycoCard Reader II device (Axis-Shield PoC AS, Norway). In addition, 7 patients from the main group underwent continuous monitoring of blood glucose (CGM) in a blind mode using an iPRO-2 continuous monitoring system (Medtronic, USA). This measurement was made during the first four days of the hospital stay; the average age of patients in this subgroup was 52.2±9.4 years and the duration of DM — 4.5±2.7 years. According to CGM, the mean glycemic parameters, the duration (percentage of time) of hyper- and hypoglycemia (i.e. >7.8 and <3.9 mmol/L), as well as the variability of glycemia in terms of the mean amplitude of glycemic excursions (MAGE) were assessed.

For **statistical processing** of the results, we used the Statistica 8.0 and MedCalc software packages. To compare quantitative data between two independent samples, the Mann–Whitney test was used; for qualitative comparison — the c^2^ and Fisher tests, for multiple intragroup comparisons in dynamics — the Friedman method (p_dyn_), for two quantitative indicators in dynamics — the Wilcoxon test, and for correlation analysis — the Spearman’s correlation. To determine the predictors of adverse outcomes, univariate and multivariate regression analyses were used in the logistic regression model. The data samples were characterized by the mean value  ±  standard deviation (M±σ). Differences were considered significant at p≤0.05.

## Results and Discussion

The course of COVID-19 and its outcomes in both groups are characterized in Table 1.

**Table 1:** The course and treatment of COVID-19 in patients without and with diabetes mellitus

Index	Control group (n=32)	Main group (n=32)	р
**Severity of pneumonia (CURB-65)**			
*risk of death* (%) (M±σ)	4.5±1.6	6.0±3.6	0.043
**CT at admission (abs. number/%)**			
*percent of lung damage:*			
less than 50	26/81.2	19/59.4	0.050
more than 50	6/18.8	13/40.6	—
**CT, 1^st^ week (abs. number/%)**			
*positive dynamics*	23/71.9	15/46.9	0.037
**Treatment of COVID-19 (abs. number/%)**			
*glucocorticoids*	10/31.2	15/46.9	0.15
*anticoagulants:*	32/100	32/100	—
enoxaparin sodium in the minimum dose	12/37.6	6/18.8	0.082
*antiviral therapy:*	32/100	32/100	—
hydroxychloroquine	24/75.0	14/43.7	0.011
lopinavir/ritonavir	3/9.4	9/28.1	0.053
umifenovir	6/18.8	9/28.1	0.28
*antibiotic therapy:*	32/100	32/100	—
azithromycin	23/71.9	20/62.5	0.30
ceftriaxone	6/18.8	10/31.2	0.49
levofloxacin	13/40.6	16/50.0	0.31
amoxiclav	11/34.4	16/50.0	0.30
others	5/15.6	3/9.4	0.35
*immunosuppressants and other immunoactive agents:*	17/53.1	25/78.1	0.032
baricitinib	2/6.2	6/18.8	0.26
human antibodies (Ig)	4/12.5	10/31.2	0.24
interferon α-2b	11/34.4	9/28.1	0.39
**Respiratory rate on admission (M±σ)**	19.0±1.5	21.0±4.1	0.005
**O_2_ saturation (%) (M±σ):**			
on admission, day 1	94.4±2.8	93.1±4.4	—
day 2	90.3±17.0	92.4±4.6	0.29
day 3	94.8±2.5	94.4±2.4	0.34
day 4	95.3±2.2	92.8±4.2	0.69
day 5	94.8±3.3	93.6±3.6	0.033
day 6	95.7±2.4	93.8±2.9	0.066
day 7	95.8±2.3	93.8±2.9	0.024
p_dyn_*	0.042	0.18	0.004
**Number of days before normalization of SрO_2_ (M±σ)**	4.1±3.8	9.8±6.8	0.0004
**Unfavorable outcome (abs. number/%):**	3/9.4	6/18.8	0.24
hospitalization in the ICU	3/9.4	6/18.8	0.24
fatal outcome	1/3.1	1/3.1	—
**Duration of treatment, bed-days (M±σ)**	14.3±3.1	17.1±4.8	0.013

* p_dyn_ — significance of the differences between values in dynamics (by Friedman).

Among patients with background DM, viral pneumonia was more severe, as evidenced by a 2.2-fold larger proportion of patients with extensive (>50%) involvement of lung tissue, according to CT results (p=0.050). There was a 1.5-fold smaller number of patients showing a rapid positive dynamics with CT during the first week of treatment (p=0.037) and a 1.3-fold higher risk of death as calculated from the CURB-65 scale (p=0.043).

The severe course of pneumonia in DM was associated with more pronounced symptoms of respiratory failure, including an increased respiratory rate at the time of admission (p=0.005). We observed a sustained decrease in blood oxygen saturation: on days 1–3 of hospital stay, SpO_2_ was similarly decreased in both groups, on days 4–7, it remained low only in patients with the combined pathology, where its normalization required a 2.4-fold longer time (p=0.0004).

The increased severity of viral pneumonia in the main group required a more aggressive therapy. As shown in Table 1, patients with COVID-19 and DM were less likely to receive hydroxychloroquine (p=0.011); instead they were prescribed modern immunosuppressors and immunoactive drugs (p=0.032) with a trend to receive combined antiviral agents (lopinavir + ritonavir, p=0.053) and larger doses of anticoagulants (p=0.082).

As a result, the duration of treatment (total bed-days) in the main group was significantly longer (p=0.013) with an insignificantly higher proportion of patients who reached the composite endpoint (hospitalization in the ICU and/or death, p=0.24).

Thus, the co-occurrence of COVID-19 and DM in patients was characterized by a greater severity and extension of viral pneumonia, a persistent decrease in blood oxygen supply (by pulse oximetry and clinical signs), and a greater need for active therapy (modern immunosuppressive, immunoactive and combined antiviral drugs, as well as higher doses of anticoagulants). In these patients, the duration of hospital stay was significantly longer.

Another area of our research was the metabolic changes and laboratory characteristics of patients with COVID-19 without and with DM, as well as their relationship with the severity of the disease and the outcomes (Table 2).

**Table 2:** Metabolic, biochemical, and coagulation parameters of patients with COVID-19 in the presence or absence of diabetes mellitus (M±σ)

Index	Control group (n=32)	Main group (n=32)	р
Blood glucose on admission (mmol/L)	4.8±0.8	8.1±2.9	0.0000
Creatinine (μmol/L):			
on admission	85.9±25.4	98.4±32.7	0.23
days 3–5	98.6±25.6	113.6±33.4	0.035
at discharge	96.2±19.1	95.2±27.9	0.56
p_dyn_*	0.43	0.020	—
Glomerular filtration rate (ml/min):			
on admission	74.7±17.97	65.5±21.8	0.10
days 3–5	64.2±17.6	55.9±19.8	0.09
at discharge	64.7±16.0	67.3±23.0	0.61
p_dyn_*	0.31	0.067	—
D-dimer (ng/ml):			
on admission	824.2±1291.8	986.4±1690.7	0.89
day 2	618.0±1020.4	421.6±632.5	0.81
days 3–5	663.8±1215.3	615.4±987.5	0.92
at discharge	59.1±111.2	31.2±106.9	0.22
p_dyn_*	0.004	0.0003	—
Day of D-dimer normalization (if abnormal at baseline)	5.6±4.5	9.0±6.3	0.035
Fibrinogen (g/L):			
on admission	5.5±1.6	6.0±1.8	0.22
days 3–5	4.4±0.9	5.2±1.2	0.013
at discharge	3.9±0.9	4.0±1.5	0.73
p_dyn_*	0.00001	0.00001	—
Day of fibrinogen normalization (if abnormal at baseline)	8.3±5.5	11.8±5.5	0.037
APTT (s):			
on admission	30.1±6.1	34.9±26.8	0.91
days 3–5	33.7±7.1	40.6±24.8	0.77
at discharge	37.0±17.4	36.9±16.1	0.55
p_dyn_*	0.004	0.23	—
PTT (s):			
on admission	12.8±1.3	13.4±3.7	0.38
days 3–5	12.4±1.6	13.6±4.1	0.064
at discharge	16.4±19.3	12.8±2.9	0.81
p_dyn_*	0.28	0.019	—
INR:			
on admission	1.1±0.1	1.1±0.3	0.51
days 3–5	1.1±0.1	1.1±0.4	0.52
at discharge	1.1±0.3	1.1±0.3	0.92
p_dyn_*	0.29	0.16	—
Day of INR normalization (if abnormal at baseline)	3.7±4.1	6.1±6.5	0.14
AT III (%):			
on admission	107.0±13.7	107.4±19.5	0.64
days 3–5	96.2±13.2	95.4±13.3	0.96
at discharge	96.1±11.7	98.0±13.2	0.64
p_dyn_*	0.001	0.058	—
Day of AT III normalization (if abnormal at baseline)	1.6±1.9	4.4±5.6	0.012
ALT (units/L):			
on admission	36.7±23.7	40.2±25.4	0.56
days 3–5	59.9±55.8	89.6±96.1	0.22
at discharge	58.1±45.6	63.9±78.8	0.91
p_dyn_*	0.002	0.017	—
AST (units/L):			
on admission	34.8±16.6	49.9±44.5	0.16
days 3–5	54.8±46.8	68.5±62.2	0.26
at discharge	37.6±25.4	43.6±29.0	0.62
p_dyn_*	0.057	0.015	—
CRP (mg/L):			
on admission	41.6±37.3	91.3±90.0	0.028
days 3–5	51.1±61.2	97.1±87.3	0.015
days 7–10	53.5±57.3	60.1±65.5	0.86
at discharge	7.3±9.9	10.3±16.7	0.50
p_dyn_*	0.001	0.00001	—
Hemoglobin (g/L):			
on admission	138.1±12.97	137.5±13.8	0.86
at discharge	125.7±16.8	125.5±16.3	0.90
p_dyn_**	0.0001	0.0003	—
Hematocrit (%):			
on admission	40.7±3.7	40.9±3.8	0.61
at discharge	37.7±4.0	37.9±4.5	0.70
p_dyn_**	0.0001	0.003	—
Leukocytes (×109/L):			
on admission	6.3±2.7	6.7±3.1	0.71
at discharge	5.1±1.9	6.2±1.6	0.012
p_dyn_**	0.021	0.63	—
ESR (mm/h):			
on admission	29.3±21.5	34.4±21.3	0.22
at discharge	16.9±10.6	26.0±20.0	0.048
p_dyn_**	0.007	0.16	—

* p_dyn_ — significance of the differences between values in dynamics by Friedman;

** — by Wilcoxon.

The levels of blood glucose in the main group, as expected, exceeded those in control (p<0.00001).

**Figure 1:**
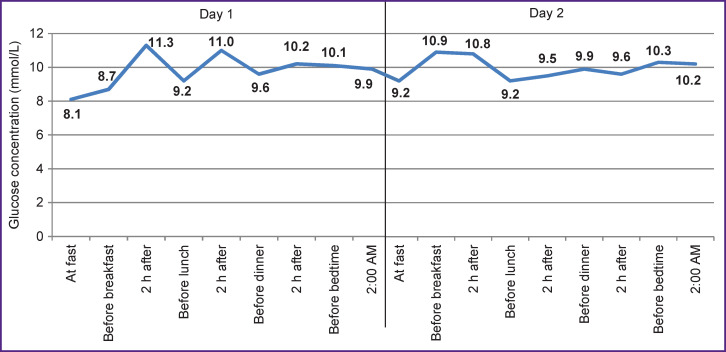
Average values of glycemic indices in patients with COVID-19 and diabetes mellitus on days 1 and 2 of hospitalization

Hyperglycemia in patients with DM persisted, especially on the first and second days of hospitalization (Figure 1) even with the aggressive glucose-lowering therapy.

**Figure 2:**
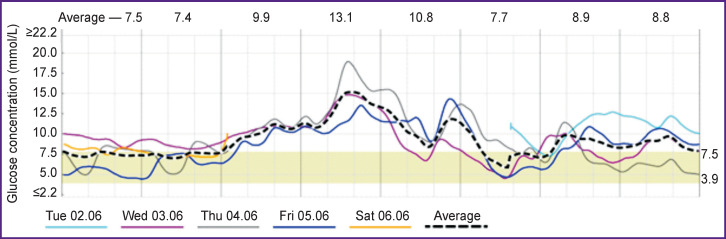
Results of continuous glucose monitoring in patient M. (main group)

The results of CGM, which was carried out during the first four days of inpatient treatment in seven patients with COVID-19 and DM, confirmed the presence of hyperglycemia for more than half of the observation period (53% of time with glycemia >7.8 mmol/L, the average glucose level 8.3±1.5 mmol/L), without pronounced variability of glycemia (MAGE 1.5±0.6 mmol/L) and frequent hypoglycemic episodes (glucose <3.9 mmol/L — 1.4% of the CGM duration). Graphical display of CGM data obtained from a patient with COVID-19 and DM is shown in Figure 2.

The development and exacerbation of hyperglycemia in SARS-CoV-2 is facilitated by specific effects of infection on carbohydrate metabolism in the form of:

1. involvement of ACE2: the enzyme serves as a functional receptor for the virus; ACE2 is expressed in the liver and pancreas, turning them into a potential target of the virus and thereby increasing hyperglycemia [31, 32];
2. activation of the transcription factor of genes encoding for pro-inflammatory cytokines (specifically, interferon-regulating factor-5) during a cytokine storm and its subsequent binding to uridine diphosphate-N-acetylglucosamine; as the latter is formed during glucose metabolism, the entire process may stimulate glucose production by a feedback mechanism [33];
3. increase in the formation of glucose due to the decomposition of deoxyhemoglobin — glycated hemoglobin by the virus [6, 34].

In turn, hyperglycemia serves as a pathogenetic factor that worsens the infection-associated outcome, including respiratory infections [30, 35]. According to previous reports [36, 37], hyperglycemia can be associated with a higher glucose level in the alveolar secretion, an increase in viral replication, impaired immune response to the virus, and aggravation of lung disorders. As with other viral infections, hyperglycemia in COVID-19 is a risk factor for death; however, an increase in HbA1c in patients without uncontrolled hyperglycemia is associated with a significantly lower risk for the patient [38].

According to our data, the level of glucose upon admission in the main group of patients correlated inversely with the parameters of blood O_2_ saturation (R=–0.42; p=0.018), which indirectly confirmed the negative effect of high hyperglycemia on the respiratory function and oxygen supply under COVID-19. In addition, for the entire cohort of patients, glycemia turned out to be an independent predictor of severe pneumonia (the CURB-65 score in the upper quartile range): odds ratio (OR) 1.02 [1.00; 1.03]; p=0.016, according to one-way regression analysis.

On the other hand, HbA1c significantly correlated only with the duration of DM (R=0.47; p=0.015), the incidence of late DM complications (R=0.41; p=0.017), and with the blood lactate level (R=0.42; p=0.018); the expected weak correlation with the level of blood glucose at admission was also noted (R=0.30; p=0.034). Thus, the level of HbA1c was consistently higher in patients with a long-term history of severe DM; it was associated with some metabolic disorders, but, unlike hyperglycemia, had no prognostic value for predicting a severe course of the disease. This result largely corroborates with the data available in the literature [38] and does not rule out that an unsatisfactory previous control of DM may have an implicit negative impact on the course of COVID-19  +  DM; this negative influence may be mediated by uncontrolled hyperglycemia at the initial stage and additional metabolic complications.

Another factor that significantly affects the course of SARS-CoV-2 infection was the functional state of the kidneys. Typically, in both groups there was a slight decrease in renal function on days 3–5 of the hospital stay followed by recovery by the time of discharge. In Table 2, these changes are more noticeable in patients with DM, e.g., the significantly higher values of creatinine compared with control after 3–5 days of therapy (p=0.035), as well as the statistically significant dynamic fluctuations (p_dyn_=0.020).

The creatinine level on days 3–5 served as one of the laboratory predictors of reaching the predefined outcome (admission to the ICU and/or death) for the pooled cohort of patients with COVID-9 in the univariate analysis: OR — 1.02 [1.00; 1.06]; p=0.011. Among patients with DM, creatinine failed to significantly predict the poor outcome (OR — 1.02 [0.99; 1.055]; p=0.083), but it was associated with a more severe pneumonia (CURB-65 scale; R=0.43; p=0.018).

For assessing damage to the liver caused by COVID-19 and insufficient therapy, the levels of hepatic transaminases was monitored over time. Like creatinine, those showed an increase by day 3–5 of hospitalization with a subsequent decrease (for ALT, p_dyn_=0.017), while the differences between the main and control groups were not statistically significant (see Table 2).

According to the literature, liver dysfunction occurs both in DM and COVID-19. In patients with combined (DM and COVID-19), the liver involvement (as it was put by Marhl et al. [39]) forms the pathological “hepatic axis”, common pathogenetic mechanisms leading to the mutual aggravation of the diseases. ALT is a sensitive marker of liver damage in both pathologies. In COVID-19, even a slight increase in ALT is considered a predictor of a severe course of this infection [27, 28, 40]. In people with DM, a slight but persistent increase in ALT could be associated with non-alcoholic fatty liver disease [39, 41].

Our results are in good agreement with the literature and, in general, confirm the significance of the ALT level for predicting unfavorable outcomes in all patients under study: in univariate analysis (OR — 1.03 [1.01; 1.06]; p=0.009). However, with a combination of COVID-19 and DM, the ALT level was not a significant predictor of the outcome. This may be due to the short duration of DM and, therefore, the absence of hepatosis in most patients. Nevertheless, the correlation analysis confirmed an increase in the severity of pneumonia (by the CURB-65 scale) with an increase in ALT level (R=0.55; p=0.001). In the main group, direct correlations of ALT with glycemic levels on the first day of treatment were also found (including that for fasting glucose, R=0.44; p=0.022); this finding does not rule out an additional deterioration of carbohydrate metabolism in the case of hepatic dysfunction and may partially mediate pneumonia aggravation.

One of the recognized common links in the pathogeneses of COVID-19 and DM is systemic inflammation, markers of which play the role of predictors of severe course of both diseases [42, 43]. According to our data, in both groups, the average CRP level was significantly increased throughout hospitalization, including the day of discharge from the hospital. This result confirms the typicality and significance of inflammatory changes in the pathogenesis of COVID-19 (see Table 2). In addition, in univariate analysis in the pooled cohort of patients, CRP was identified as a statistically significant predictor of the outcome (OR — 1.02 [1.00; 1.04]; p=0.003). The combined pathology though was characterized by a greater activity and sustained pattern of inflammation. Thus, the level of CRP in the main group was significantly higher than that in control, both at admission (p=0.028) and 3–5 days after hospitalization (p=0.015). Initially, ESR increased similarly in both observation groups (p=0.22), but in dynamics, it decreased more slowly in the presence of DM, due to which, at discharge, ESR significantly exceeded the control values (p=0.048) and the upper limit of the norm.

Another central pathogenetic factor complicating the course of COVID-19 is coagulopathy that manifests by hypercoagulability and a high risk of venous, arterial, and microvascular thrombosis [29]. Apparently, the likelihood of thrombosis in SARS-CoV-2 infection exceeds the similar risks in other acute infectious diseases. This is explained by a specific effect of this virus on blood coagulation processes, mediated by particularly high inflammation, production of pro-inflammatory cytokines, imbalance of RAAS mediators, and direct contact between the virus and the endothelium [24, 29, 44–46].

According to our data, hypercoagulability and high thrombogenic activity are characteristic of the entire cohort of SARS-CoV-2 infected subjects (see Table 2). In both studied groups, the average levels of D-dimer and fibrinogen significantly exceeded the norm, at least at the beginning of the hospital period; later on, the observed lengthening of APTT was less than expected (considering that anticoagulant therapy was used in 100% of cases, see Materials and Methods).

In a comparative assessment of blood coagulation indices, we noted significant differences between the main and control groups, indicating a greater severity and duration of coagulopathies in patients with concomitant DM. Thus, with DM, the abnormally high levels of AT III (p=0.012), fibrinogen (p=0.037), and D-dimer (p=0.035) remained significantly longer. On top of that, a greater degree of hyperfibrinogenemia was noted, especially on days 3–5 (p=0.013), the PTT decreased over time (p=0.019) and no APTT lengthening occurred (p=0.23). In addition, according to univariate regression analysis, the level of fibrinogen at admission significantly predicted the adverse outcomes in the pooled cohort of patients (OR — 2.08 [1.27; 3.40]; p=0.0007).

In order to identify the most significant predictors of reaching the defined outcome (hospitalization in the ICU and/or death), we carried out a step-by-step multivariate regression analysis of clinical and laboratory parameters identified in univariate analysis as significant or close to significant predictors. We found that for the pooled cohort of patients (with and without DM), fibrinogen and CRP had predictive values (OR — 2.3 [1.11; 4.76] and 1.02 [1.004; 1.045], respectively; p=0.00025), and for the DM subgroup that was fibrinogen (OR — 2.6 [1.09; 6.22]; p=0.003).

The obtained data confirm the role of coagulopathy in the pathogenesis and clinical course of SARS-CoV-2 infection, its increased severity and duration in concomitant DM, as well as its contribution to the development of poor outcomes. Considering the results of one- and multivariate analysis, it cannot be ruled out that the COVID-19 associated liver and kidney dysfunctions may manifest as coagulopathies.

## Conclusions

The results of the study allowed us to draw the following conclusions:

1. The presence of concomitant DM in patients with COVID-19 is associated with a greater severity and extension of pneumonia, a sustained decrease in oxygen supply, an increased need for modern immunosuppressive, immunoactive, and combined antiviral drugs, as well as in high doses of anticoagulants, which in total leads to a significant lengthening of the hospital stay.
2. High hyperglycemia in patients with SARS-CoV-2 infection and DM is associated with a decrease in blood oxygen saturation, a more severe respiratory failure, and an aggravated course of pneumonia. The increased level of HbA1c is less correlative with the pneumonia severity. Previous imbalanced diabetes may have an implicit negative effect on the course of COVID-19 + DM, which may be mediated by uncontrolled glycemia and aggravated metabolic disorders.
3. Patients with COVID-19 are characterized by impaired renal and hepatic functions, which may worsen in the first days of hospitalization; the levels of creatinine and AST are interrelated with the risk of unfavorable outcomes of the disease (hospitalization in the ICU and/ or death). In DM, these dysfunctions can worsen and lead to high creatinine levels.
4. With COVID-19, pronounced and persistent systemic inflammation occurs, which decreases but does not disappear by the end of the hospital period. The presence of DM in those patients additional increases the systemic inflammation. Abnormally high CRP levels predict a severe course of COVID-19.
5. Patients with COVID-19 are characterized by hypercoagulability, which is accompanied by a pronounced and sustained increase in the levels of D-dimer and fibrinogen in the blood. The severity of coagulopathies and the time needed for normalization of the coagulogram were greater in those with DM. Fibrinogen level can serve an independent predictor of adverse outcomes of COVID-19, especially in patients with DM.
